# Reduction of solid tumors by senescent cell immunization

**DOI:** 10.1186/s12967-025-07393-3

**Published:** 2025-11-28

**Authors:** Thomas E. Ichim, Gilberto Lopes, Robert Reznik, Vladyslav Bykoriz, Christian A. Fortunati, Karenjan A. Pascual, Boris Minev, Roman A. Ramos, Anil Bajnath, Emma Lin, Joyce Hu, Francesco M. Marincola, Armin Rath, Boris N. Reznik

**Affiliations:** 1 Immorta Bio Inc., Miami, FL and San Diego, CA USA; 2 Department of Medical Oncology, Miller School of Medicine, University of Miami, Miami, FL USA; 3 Department of Radiation Oncology, Cedars Sinai, Los Angeles, Ca USA; 4 Department of Radiation Oncology, University of California San Diego, San Diego, CA USA; 5 Institute for Human Optimization, Hanover, MD USA; 6 Department of Medicine, George Washington University, Washington, DC USA; 7 Biocenturium LLC, San Diego, CA USA; 8 Translational and Advanced Medicine (TAM) Biosciences, Nashville, TN USA

## Abstract

**Background:**

Immunologically mediated clearance of senescent cells has been demonstrated in several model systems. Given increasing evidence for these cells promoting tumor pathology and immune escape, we sought to examine whether a vaccine against senescent cells can lead to tumor regression.

**Materials and Methods:**

A senolytic dendritic cell (DC) immunotherapy (“SenoVax™”) was created by pulsing DCs with lysate from in vitro generated syngeneic senescent fibroblasts. Prophylactic and therapeutic activity of SenoVax on tumor growth and metastasis was assessed in the Lewis Lung Carcinoma (LLC) model. The immunogenicity of SenoVax™ was measured using cytotoxicity, proliferation, and cytokine assays. Adoptive transfer of lymphocytes from vaccinated mice into naïve mice was performed in a prophylactic tumor challenge model. Assessment of plasma senescence associated biomarkers IL-11, IL-6, IL-23 receptor, and YLK-40 was performed by ELISA. Synergy of SenoVax™ with immune checkpoint inhibitors and the universality of the vaccine’s effects against other tumors was assessed. Furthermore, induction of autoimmunity was assessed by complement activation and autoantibody formation.

**Results:**

SenoVax™ was created by pulsing DC with cell lysate from senescent fibroblasts, producing DCs that expressed co-stimulatory molecules, stimulated T cell proliferation, and expressed the senescence antigen p16. SenoVax™ induced prophylactic and therapeutic tumor regression in LLC primary and metastatic murine tumor models. T cell proliferative and cytokine recall responses towards senescent cells but not to control stromal cell pulsed DCs were detected in vaccinated mice. Additionally, reduction in senescence associated biomarkers IL-11, IL-6, IL-23 receptor, and YLK-40 were observed. Adoptive transfer experiments revealed a role for CD8+ T cells in transplanting protection. When SenoVax™ was administered in combination with anti-PD-L1 or anti-CTLA-4 antibodies, the data showed synergistic effects in reducing tumor growth. SenoVax™ also demonstrated reduction of GL281 glioma, Pan01 pancreatic cancer, and 4T1 breast cancer cell growth. No significant activation of complement or induction of autoantibodies was observed.

**Conclusion:**

Vaccination with DC pulsed senescent cells resulted in reduction of tumor growth in a CD8+ T cell and interferon gamma-associated manner in lung cancer as well as other tumor models. The data provide mechanistic support for advancement of senolytic immunotherapy as a novel form of cancer therapy.

## Introduction

Lung cancer represents a significant cause of morbidity and mortality with ~1.8 million annual deaths, making it the leading cause of cancer death in the US. It is accepted that non-small cell lung cancer (NSCLC), which includes histological subtypes such as adenocarcinoma, squamous cell carcinoma, and large cell carcinoma, is responsible for about 85% of pulmonary neoplasia [1, 2]. Despite the effectiveness of surgery in patients with resectable early-stage or locally advanced tumors, a significant proportion, up to 70%, of surgical patients have tumor recurrence [3]. Unfortunately, the majority of patients are diagnosed with late-stage disease for which the average 5-year survival rate is approximately 15% [4].

Therapeutic advances in treatment of lung cancer have included targeted therapies, which are available for patients with mutations in oncogenes such as ROS Proto-Oncogene 1 (ROS1), epidermal growth factor receptor (EGFR), anaplastic lymphoma kinase (ALK), and B-raf proto-oncogene (BRAF) [5–8]. Unfortunately, a significant number of patients do not harbor such targetable mutations, and development of treatment resistance remains a significant issue for long term and durable control. Immunotherapy in the form of checkpoint inhibitors is gaining interest as a therapeutic modality for NSCLC. For example, antibody mediated blockade of the immune inhibitory programmed cell death protein 1 (PD-1) and its ligand PD-L1 has resulted in significantly extended patient survival, which was the basis for regulatory approval of these types of therapeutic agents [9]. Numerous mechanisms of therapeutic activity have been described for checkpoint inhibition including prevention of T cell exhaustion [10–12], enhancement of number and activity of tumor-infiltrating T cells with stem cell-like properties [13], and modulation of dendritic cell activation [14]. Unfortunately, primary and developed resistance to checkpoint inhibitors remains a significant hurdle, thereby resulting in tumor recurrence in majority of cases [15].

Tumors possess numerous immune suppressive mechanisms that subvert immunotherapy. Mechanisms of tumor escape from immunological pressure, whether naturally occurring or therapeutically-induced include T regulatory cells [16–18], type 2 macrophages [19], type 2 neutrophils [20], myeloid suppressor cells [21], cancer associated fibroblasts [22], and immature dendritic cells [23]. Recent studies have shown that senescent cells surrounding the tumor microenvironment can suppress immunity to various tumors [24, 25].

One of the natural functions of the immune system is clearance of senescent cells. This has led to one of the theories of aging, in which the reduction of immune activity by senescence allows for accelerated aging by accumulation of senescent cells that lack a mechanism for clearance. Accordingly, stimulation of the immune system is one strategy that can be used to increase the clearance of senescent cells for preventing, alleviating, or lessening the burden of disease. This concept has been demonstrated by studies performed in which peptide, protein, and nucleic acid vaccination has resulted in immunity to senescent cells [26]. One strategy for inducing a potent immune response against senescent cells involves polyvalent vaccination using whole cell lysate comprising the antigenic source (i.e., senescent cells) and immune stimulatory cells (i.e., DCs). A therapeutic approach involving a polyvalent vaccine possesses the advantage of stimulating multiple clones of T cells and B cells, thus addressing the heterogeneity of tumors and reducing the possibility of treatment resistance [27]. In cancer therapeutics, previous studies have used whole cells or cell lysates as a means of stimulating immunity to cancer cells and cells in the tumor microenvironment [28, 29].

In the present study, a multivalent immunotherapeutic approach directed toward senescent cells was created, involving administration of DCs pulsed with lysate from senescent fibroblasts, creating an autologous vaccine termed “SenoVax™.” This report describes the generation of the SenoVax™ approach to vaccinating against senescent cells and demonstrates the immunological responses against tumors that ensue using this approach. These preclinical studies address the clinical relevance of vaccinating against senescence-associated antigens as a potential clinical strategy for treating cancer.

## Materials and methods

### Animals

Female BALB/c and C57BL/6 mice (6–8 weeks old) were purchased from Jackson Laboratory (Bar Harbor, ME, USA) and housed under specific pathogen-free conditions at BioCentrium LLC (San Diego, CA, USA). Animals were maintained on a 12-hour light/dark cycle with ad libitum access to food and water. All experimental procedures were approved by the Institutional Animal Care and Use Committee (IACUC) at BioCentrium LLC. and conducted in accordance with the National Institutes of Health guidelines for the care and use of laboratory animals.

### Cell lines and culture

Lewis Lung Carcinoma (LLC), GL281 glioma, Pan01 pancreatic cancer, and 4T1 breast cancer cell lines were obtained from the American Type Culture Collection (ATCC, Manassas, VA, USA). Cells were cultured in Dulbecco’s Modified Eagle Medium (DMEM; Gibco, Thermo Fisher Scientific, Waltham, MA, USA) supplemented with 10% fetal bovine serum (FBS; Gibco), 100 U/mL penicillin, and 100 µg/mL streptomycin (Gibco) at 37 °C in a humidified 5% CO₂ atmosphere. Primary murine fibroblasts were isolated from the skin of C57BL/6 mice using standard enzymatic dissociation protocols and cultured in the same conditions.

### Induction of cellular senescence

Senescent fibroblasts were generated in vitro by treating primary C57BL/6 fibroblasts with doxorubicin (Sigma-Aldrich, St. Louis, MO, USA) at a concentration of 0.2 µM for 48 hours, followed by a 7-day recovery period in fresh medium. Senescence was confirmed by assessing senescence-associated β-galactosidase (SA-β-gal) activity using a commercial kit (Cell Signaling Technology, Danvers, MA, USA), and expression of p16 and p21 by ELISA (Abcam, Cambridge, UK). Interleukin-6 (IL-6) secretion, indicative of the senescence-associated secretory phenotype (SASP), was quantified in culture supernatants by enzyme-linked immunosorbent assay (ELISA; R&D Systems, Minneapolis, MN, USA).

### Generation of SenoVax™ dendritic cell vaccine

Bone marrow-derived dendritic cells (DCs) were generated from mice syngeneic to the tumor. Briefly, bone marrow cells were flushed from femurs and tibias, and mononuclear cells were isolated by centrifugation over a Ficoll-Paque gradient (GE Healthcare, Chicago, IL, USA). Cells were cultured in RPMI-1640 medium (Gibco) supplemented with 10% FBS, 20 ng/mL granulocyte-macrophage colony-stimulating factor (GM-CSF; PeproTech, Rocky Hill, NJ, USA), and 10 ng/mL interleukin-4 (IL-4; PeproTech) for 7 days. DC phenotype was confirmed by flow cytometry using antibodies against CD40, CD80, and CD86 (BD Biosciences, San Jose, CA, USA). DC functionality was assessed by mixed lymphocyte reaction (MLR), where DCs were co-cultured with allogeneic T cells from BALB/c mice, and proliferation was measured by ^3 H-thymidine incorporation (PerkinElmer, Waltham, MA, USA).To generate SenoVax™, senescent fibroblasts were lysed by three freeze-thaw cycles in liquid nitrogen and a 37 °C water bath. Lysates were centrifuged at 10,000 × g for 10 minutes to remove debris, and the supernatant was used to pulse DCs at a ratio of 1:10 (DC:lysate protein equivalent) for 24 hours. Pulsed DCs were assessed for viability using Trypan Blue exclusion (Sigma-Aldrich) and for p16 uptake by flow cytometry using an anti-p16 antibody (Abcam). SenoVax™ DCs were washed and resuspended in phosphate-buffered saline (PBS) for in vivo administration.

### Tumor models and vaccination protocols

For the Lewis Lung Carcinoma (LLC) model, 1 million cells were injected subcutaneously into the flank of C57BL/6 mice to establish primary tumors or intravenously via the tail vein to induce lung metastases. For prophylactic studies, mice received a single subcutaneous injection of 1 million SenoVax™ DCs or control DCs (pulsed with non-senescent fibroblast lysate) 7 days prior to tumor inoculation. For therapeutic studies, mice were inoculated with LLC cells, and 1 week later, received subcutaneous injections of 500,000, one million or two million cells weekly for 3 weeks. Tumor volume was measured using calipers and calculated as (length × width(2)/2. Lung metastases were quantified by counting visible nodules on the lung surface after euthanasia and staining with India ink. For other tumor models (GL281 glioma, Pan01 pancreatic cancer, and 4T1 breast cancer), 500,000 cells were injected subcutaneously into C57BL/6 (GL281, Pan01) or BALB/c (4T1) mice. SenoVax™ (one million DCs) was administered subcutaneously 7 days post-tumor inoculation, and tumor growth was monitored as described.

### Combination with checkpoint inhibitors

To assess synergy with immune checkpoint inhibitors, mice bearing LLC tumors, one week post inoculation, were treated with SenoVax™ (1 × 10(6) DCs, weekly for 3 weeks) alone or in combination with anti-PD-L1 (clone 10F.9G2, BioXCell, Lebanon, NH, USA) or anti-CTLA-4 (clone 9D9, BioXCell) antibodies. Antibodies were administered intraperitoneally at 200 µg/dose every 3 days for a total of 4 doses, starting concurrently with SenoVax™ administration. Tumor growth was monitored as described.

### Immunological assays

T Cell Proliferation and Cytokine Production: Splenocytes from vaccinated or control mice were co-cultured with SenoVax™, control DCs, or DCs pulsed with non-senescent fibroblast lysate (1:10 stimulator:responder ratio) for 72 hours. T cell proliferation was measured by 3 H-thymidine incorporation. Supernatants were collected and analyzed for interferon-gamma (IFN-γ) and granzyme B by ELISA (R&D Systems).

Cytotoxicity Assay: Plasma from vaccinated or control mice was purified using a Protein A column (Thermo Fisher Scientific) to isolate immunoglobulins. Senescent or non-senescent fibroblasts were incubated with purified immunoglobulins in the presence or absence of complement (Cedarlane Labs, Burlington, ON, Canada). Cytotoxicity was assessed using a lactate dehydrogenase (LDH) release assay (Promega, Madison, WI, USA).

Adoptive Transfer: Splenocytes from SenoVax™-vaccinated mice were enriched for CD8+ T cells, CD4+ T cells, or CD19+ B cells using magnetic-activated cell sorting (MACS; Miltenyi Biotec, Bergisch Gladbach, Germany). Recipient C57BL/6 mice were treated with cyclophosphamide (100 mg/kg, Sigma-Aldrich) 24 hours prior to adoptive transfer of 1 × 10(7) enriched cells via tail vein injection. One day later, mice were challenged with 1 × 10(6) LLC cells subcutaneously, and tumor growth was monitored.

### Senescence-associated Biomarker analysis

Plasma was collected from vaccinated and control mice at 7, 14, and 21 days post-vaccination. Concentrations of IL-11, IL-6, IL-23 receptor, and YKL-40 were quantified by ELISA (R&D Systems) according to the manufacturer’s instructions.

### Safety and autoimmunity assessment

For toxicity studies, C57BL/6 mice received SenoVax™ (1 × 10^6 DCs, weekly for 4 weeks) and were monitored for 30 (acute) or 90 (chronic) days. Hematological and biochemical parameters were assessed using a VetScan VS2 analyzer (Zoetis, Parsippany, NJ, USA). Complement activation was evaluated by measuring C3a and C5a levels in plasma using ELISA (Abcam). Autoantibody formation was assessed by screening plasma for anti-nuclear antibodies (ANA) and anti-double-stranded DNA (dsDNA) antibodies using commercial ELISA kits (Abcam).

### Statistical analysis

Data were analyzed using GraphPad Prism (version 9.0, GraphPad Software, San Diego, CA, USA). Comparisons between groups were performed using two-tailed Student’s t-tests or one-way ANOVA with Tukey’s post-hoc test for multiple comparisons. Tumor growth curves were analyzed using two-way ANOVA with Sidak’s multiple comparisons test. A p-value < 0.05 was considered statistically significant.

## Results

### Generation of SenoVax™, a dc vaccine comprising senescence-associated antigens

**Fig. 1:**
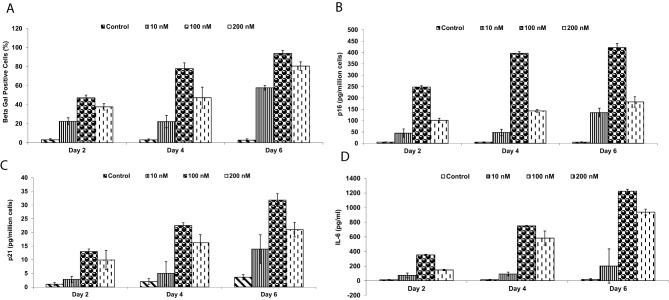
Evaluation of senescent cells generated by doxorubicin treatment. C57BL/6 fibroblasts were treated with the indicated concentrations of doxorubicin for the indicated timepoints, followed by a 7-day recovery period in fresh medium. ELISA was used to quantify (**A**) Senescence associated beta gal, (**B**) p16; (**C**) p21; and (**D**) IL-6. Assessments were performed by ELISA (data are means ± S.D; *n* = 10) Evaluation of senescent cells generated by doxorubicin treatment. C57BL/6 fibroblasts were treated with the indicated concentrations of doxorubicin for the indicated timepoints, followed by a 7-day recovery period in fresh medium. elisa was used to quantify (**A**) senescence associated beta gal, (**B**) p16; (**C**) p21; and (**d**) IL-6. Assessments were performed by elisa (data are means ± S.D; *n* = 10)

To generate a polyvalent vaccine targeting senescent cells that has relevance in cancer, we utilized fibroblasts as the cellular source, in part because numerous tumors are surrounded by senescent fibroblasts [24, 30–41]. Initial experiments were directed towards inducing cellular senescence in murine C57BL/6 fibroblasts and evaluating markers of senescence in these cells. Various inducers of senescence were evaluated, of which doxorubicin treatment showed to be the most reproducible in terms of induction of senescence-associated beta gal expression (Fig. 1a), p16 (Fig. 1b), and p21 (Fig. 1c). Additionally, a classical feature of senescent cells is the senescent associated secretory phenotype (SASP), which is comprised of inflammatory proteins such as interleukin-6 (IL-6). This cytokine has been reported to be associated with various pathological features of cancer such as metastasis, immune evasion and angiogenesis [42]. Expression of IL-6 was also induced by treatment with the senescence-inducing doxorubicin protocol (Fig. 1d).

**Fig. 2:**
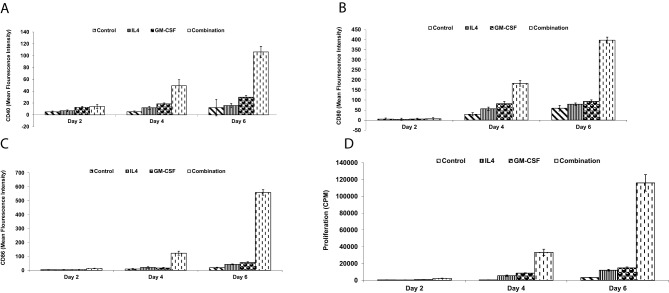
Generation of dendritic cells. Bone marrow mononuclear cells were cultured for the indicated time points in IL-4 (10 ng/ml), GM-CSF (20 ng/ml) or the combination. Expression of CD40 (**a**), CD80 (**b**) or CD86 (**c**) was assessed by flow cytometry and expressed as mean fluorescent intensity. Allostimulatory activity was assessed by culture with allogeneic BALB/c responding cells and proliferation was assessed. (data are means ± S.D; *n* = 10)

Dendritic cells (DC) are the most potent antigen presenting cells, as well as the only cell possessing the unique ability to activate naïve T cells [43]. In order to generate DC, bone marrow mononuclear cells were harvested from C57BL/6 mice and cultured in GM-CSF and IL-4 for 7 days. Flow cytometry was conducted to confirm a DC phenotype based on expression of co-stimulatory molecules CD40 (Fig. 2a), CD80 (Fig. 2b) and CD86 (Fig. 2c). The ability of the bone marrow-derived DC to incite a mixed lymphocyte reaction was assessed, which demonstrated allo-stimulatory activity of DC (Fig. 2d). The DC therefore possessed function and phenotype that was consistent with published literature on DC.

**Fig. 3:**
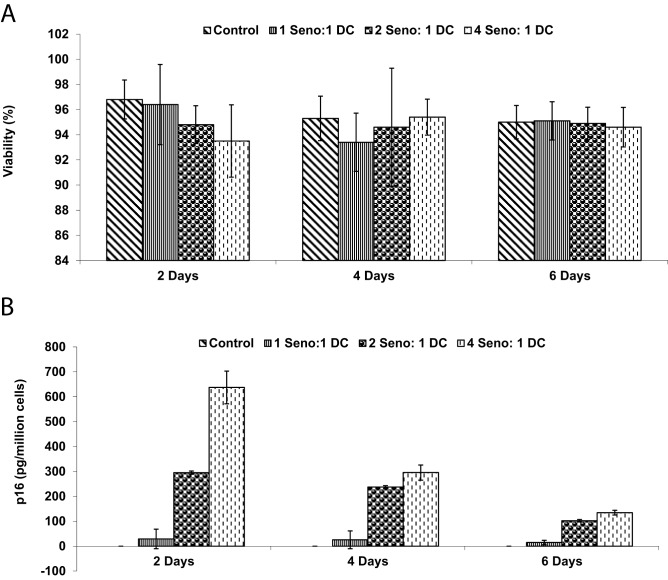
Post-pulsing properties of dc. Dendritic cells were pulsed for the indicated timepoints with senescent cell lysates obtained from concentrations of senescent fibroblast lysates. Assessment of viability was performed by trypan blue staining (**a**) and concentration of p16 was performed by ELISA (**b**). (data are means ± S.D; *n* = 10)

In order to generate a polyvalent immunotherapy, dendritic cells were pulsed with senescent fibroblast lysate. Viability of DC after pulsing with lysate (Fig. 3 (a)), as well as engulfment of p16 (b) was assessed. It was found that no significant effect on viability occurred and that p16 protein was found in the cells.

### Reduction of lewis lung cancer growth by SenoVax™

The Lewis lung carcinoma (LLC) model of lung cancer involves transplanting the LLC cell line established from the lung of a C57BL/6 mouse into a syngeneic animal [44]. This model for preclinical experimentation has been used for testing numerous cancer drugs such as vinorelbine and carboplatin and molecularly targeted agents such as sunitinib and erlotinib [45–49].

**Fig. 4:**
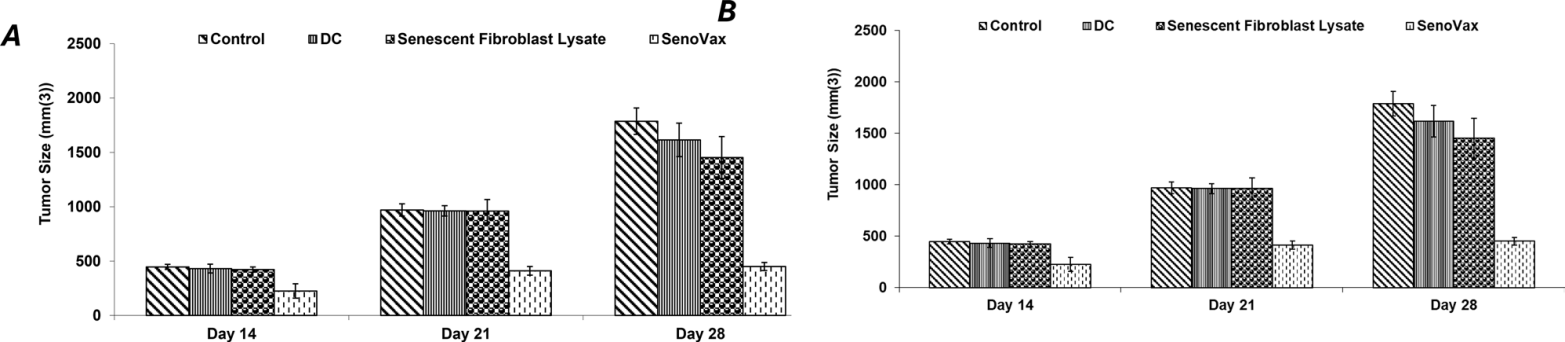
Prophylactic suppression of Lewis lung carcinoma by administration of SenoVax. Female C57/BL6 mice (6–8 weeks of age) were subcutaneously implanted with 500,000 Lewis lung carcinoma cells. Mice were treated with one injection of SenoVax 7 days prior to tumor inoculation (**a**) or three injections (**b**) given 7 days prior to tumor inoculation and once weekly. Cohorts of mice received the following treatments: a) Saline; b) Syngeneic dendritic cells (500,000) generated by 7-day culture; c) senescent fibroblast extract (equivalent to 1 million cells); and d) dendritic cells pulsed with senescent fibroblast extract (2 senescent cells to 1 dendritic cell) (SenoVax). Tumors were measured by calipers on the indicated day, and tumor size was expressed as mm(3). (data are means ± S.D; *n* = 10)

Dendritic cells pulsed with senescent cell lysate were administered subcutaneously into the LLC model to ascertain the effects of the vaccine to prophylactically suppress growth of LLC. As seen in Fig. 4, SenoVax™ suppressed tumor growth in both single administration (a) and multiple administrations (b), with multiple administrations possessing superior therapeutic effect.

**Fig. 5:**
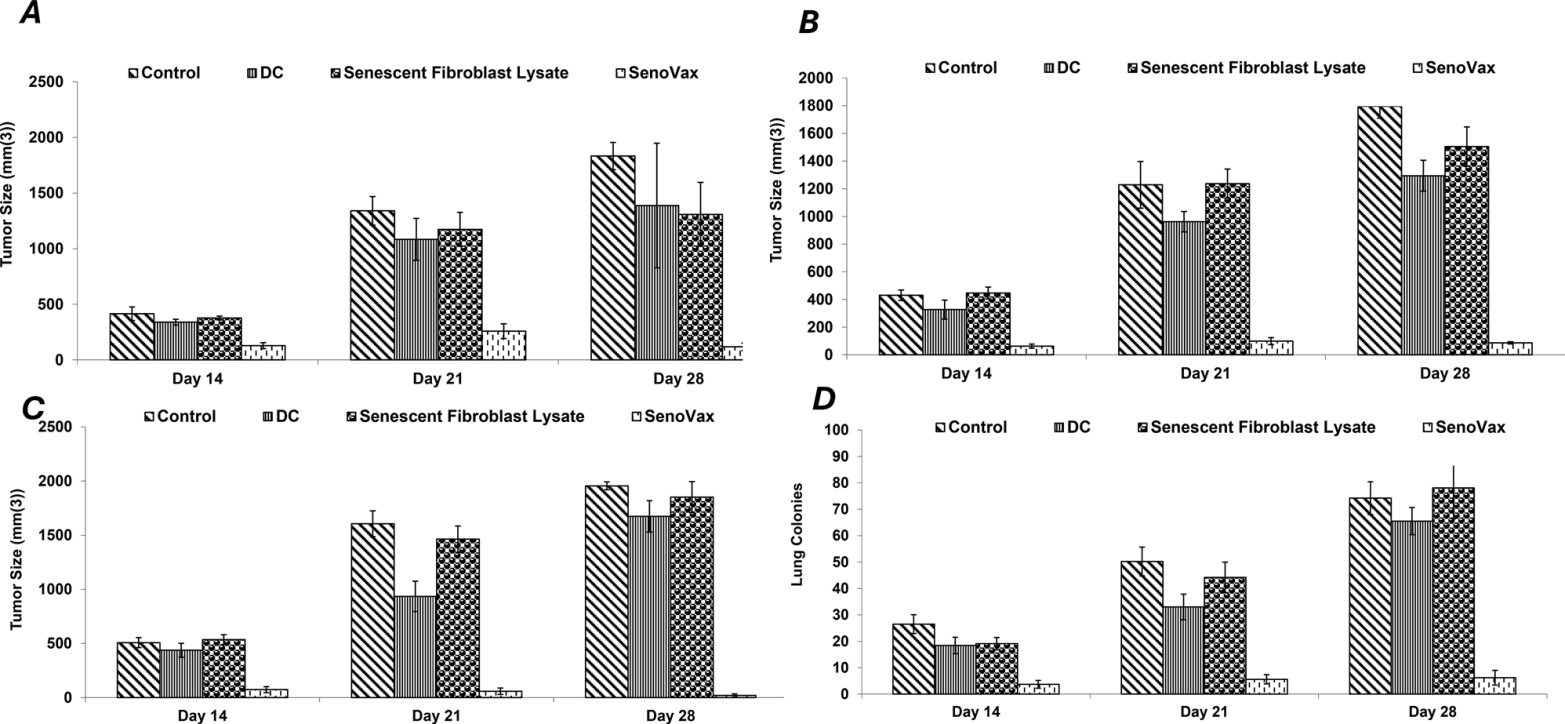
Therapeutic reduction of Lewis lung carcinoma by administration of SenoVax. Female C57/BL6 mice (6–8 weeks of age) were implanted with 500,000 Lewis lung carcinoma cells. After a week, therapeutic cells were administered at: 500,000 subcutaneously (**a**), 1 million subcutaneously (**b**), 2 million subcutaneously (**c**), and 1 million intravenously (**d**). Therapeutic injections were administered subcutaneously of 500,000 cells and comprised of (**a**) Saline; (**b**) Syngeneic dendritic cells (500,000) generated by 7-day culture; (**c**) Senescent fibroblast extract (equivalent to 1 million cells); and (**d**) dendritic cells pulsed with senescent fibroblast extract (2 senescent cells to 1 dendritic cell) (SenoVax). Tumors were measured by calipers on the indicated days and tumor size was expressed as mm(3) (**A-C**) or assessed for lung colony formation on day when mice were sacrificed. Tumors were measured by calipers on the indicated day and tumor size was expressed as mm(3). (data are means ± S.D; *n* = 10)

In order to assess therapeutic effects of SenoVax™ administration after tumor inoculation, 500,000 (Fig. 5 (a)), 1 million (b) or 2 million (c) SenoVax™ DC were subcutaneously administered 2 weeks after tumor inoculation. Suppression of tumor growth was observed. Furthermore, SenoVax™ administration was capable of suppressing lung metastasis when LLC cells were administered intravenously and the lungs were evaluated for metastatic colonies (Fig. 5 (d)).

### Mechanism of suppression of tumor growth by SenoVax™

**Fig. 6:**
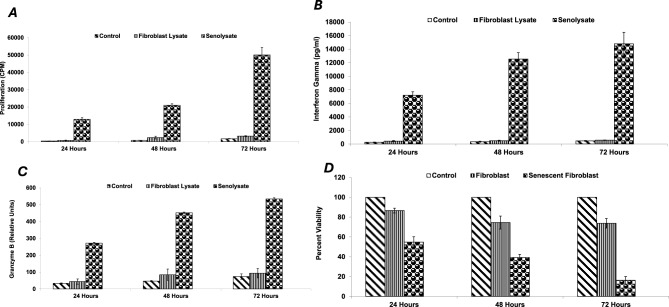
Recall responses of immunized mice to senescent cells. Splenocytes from SenoVax-immunized mice were harvested and cultured with stimulator cells that had been pulsed with lysate from senescent fibroblasts to assess the following: T cell proliferation (**a**), IFN-gamma production (**b**), granzyme B production (**c**), and antibody mediated cytotoxicity (**d**). Parellel experiments were conducted using splenocytes form non-immunized mice (control) and mice that received senescent fibroblast lysate (no DC). (data are means ± S.D; *n* = 10)

Recall response to senescent fibroblast lysate pulsed dendritic cells was assessed by culturing cells from unimmunized mice (control) or SenoVax™ immunized mice with stimulator cells containing senescent lysate. Dendritic cells alone, or dendritic cells pulsed with lysates from normal fibroblasts did not stimulate significant recall response, whereas dendritic cells pulsed with lysates of senescent fibroblasts stimulated T cell proliferation (Fig. 6 (a)), interferon gamma (b), and Granzyme B (c). To assess for generation of antibodies cytotoxic to senescent cells, immunoglobulin was concentrated using Protein A column and added to control or senescent fibroblasts in vitro. Only plasma from SenoVax™ immunized mice could induce cytotoxicity selectively to the senescent cells. Cytotoxicity was complement dependent (d).

**Fig. 7:**
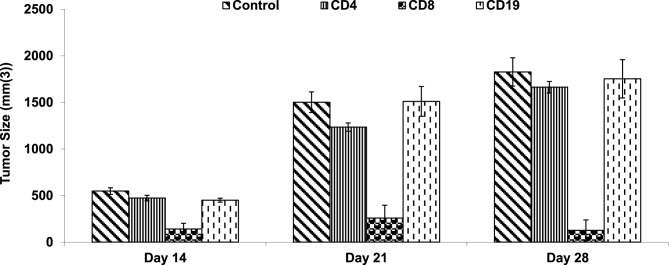
Immunological effects of SenoVax are transferred by CD8 T cells. Female C57/BL6 mice (6–8 weeks of age) were implanted with 500,000 lewis lung carcinoma cells. Splenocytes from SenoVax™-vaccinated mice were enriched for CD8+ T cells, CD4+ T cells, or CD19+ B cells using magnetic-activated cell sorting (MACS; Miltenyi Biotec, Bergisch Gladbach, Germany). Recipient C57BL/6 mice were treated with cyclophosphamide (100 mg/kg, Sigma-Aldrich) 24 hours prior to adoptive transfer of 1 × 10(7) enriched cells via tail vein injection. One day later, mice were challenged with 1 × 10(6) LLC cells subcutaneously, and tumor growth was monitored. Tumors were measured by calipers on the indicated day and tumor size was expressed as mm(3). (data are means ± S.D; *n* = 10)

To assess therapeutic populations capable of inducing tumor regression, we performed adoptive transfer experiments. Initial experiments using nondepleting conditions did not yield reduction in tumor growth. Accordingly, we used a mild cyclophosphamide regimen to create “space” for adoptively transferred lymphocytes. Accordingly, it was found that transfer of CD8 T cells but not CD4 or CD19 was capable of inducing protection from LLC tumor cells (Fig. 7). This implies a T cytotoxic mediated therapeutic activity.

**Fig. 8:**
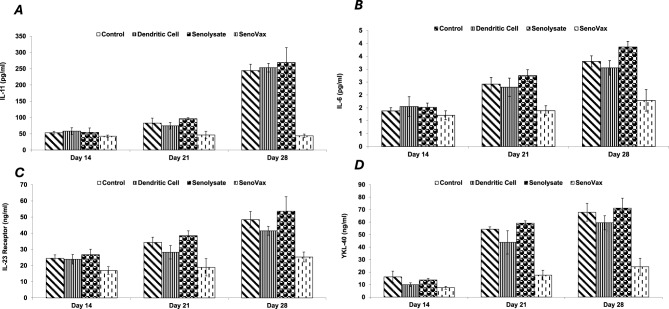
SenoVax Immunization Reduces Concentrations of Plasma Senescence Associated Proteins. Anticoagulant treated blood was drawn from mice on the indicated day and plasma was obtained by centrifugation. Assessment of the indicated cytokines was performed by enzyme linked immunosorbent assay. Data are means ± S.D; n=10

Despite induction of tumor regression, as well as in vivo generation of antibody and T cell responses, the question remained whether SenoVax™ immunization was able to induce an in vivo reduction of senescent cell burden. Accordingly, we used concentration of plasma senescence associated biomarkers as a surrogate for quantification of senescent cells. A significant time dependent reduction in IL-11 (Fig. 8a), IL-6 (Fig. 8b), IL-23 receptor (Fig. 8c), and YLK-40 (Fig. 8d) proteins was observed in immunized mice but not in controls.

### Therapeutic implications

**Fig. 9:**
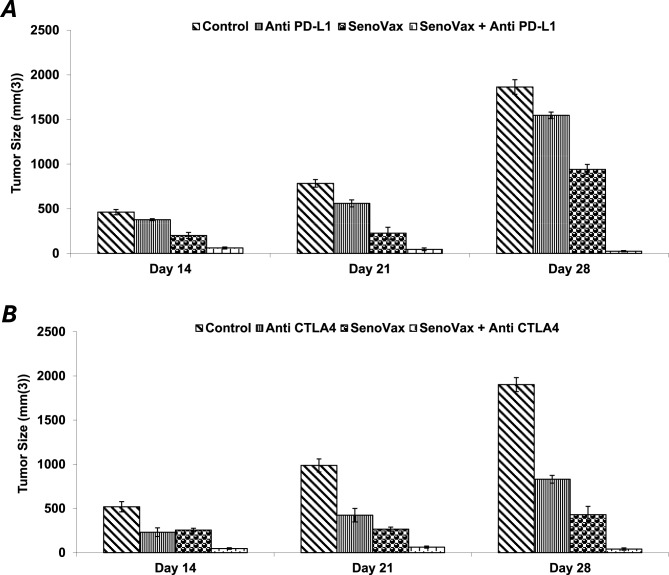
SenoVax synergizes with immune checkpoint inhibitors. Mice bearing LLC tumors, one week post inoculation, were treated with SenoVax™ (1 × 10(6) DCs, weekly for 3 weeks) alone or in combination with anti-PD-L1 (clone 10F.9G2, BioXCell, Lebanon, NH, USA) or anti-CTLA-4 (clone 9D9, BioXCell) antibodies. Antibodies were administered intraperitoneally at 200 µg/dose every 3 days for a total of 4 doses, starting concurrently with SenoVax™ administration. Tumors were measured by calipers on the indicated day, and tumor size was expressed as mm(3). (data are means ± S.D; *n* = 10)

Checkpoint inhibitors have revolutionized cancer therapy in terms of increasing survival and quality of life. Numerous novel cancer therapies are assessed together with checkpoint inhibitors to determine potential synergy. Accordingly, given the mechanism of action of SenoVax™ involves breaking of tolerance to senescent tissues, and that checkpoint inhibitors facilitate this, we assessed combination of murine anti-CTLA-4 and anti-PD-1 ligand (PD-L1) together with SenoVax™ immunization. In both cases, enhancement of antitumor effect was observed (Fig. 9).

**Fig. 10:**
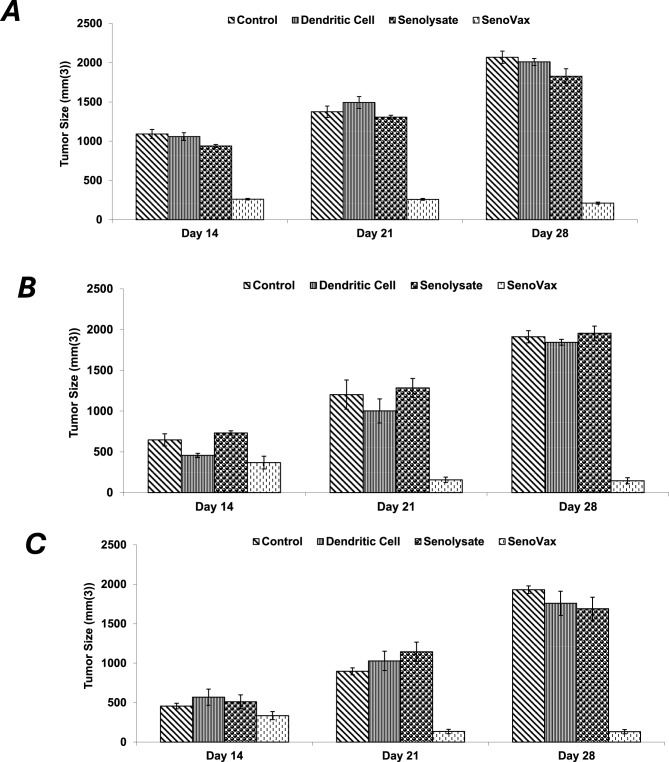
Efficacy of SenoVax in histologically unrelated tumors. 500,000 cells were injected subcutaneously into C57BL/6 (GL281, Pan01) or BALB/c (4T1) mice. SenoVax™ (one million cells or controls) was administered subcutaneously 7 days post-tumor inoculation. Tumors were measured by calipers on the indicated days, and tumor size was expressed as mm(3). (data are means ± S.D; *n* = 10)

Given the therapeutic concept behind SenoVax™ is that tumor regression occurs as a result of reduced senescent cell burden, and that tumors are associated with surrounding senescent cells, it is logical to believe that SenoVax™ should possess tumor inhibitor activity in other tissues. Immunization with SenoVax™ was able to induce regression of GL281 glioma (Fig. 10a), Pan01 pancreatic cancer (Fig. 1^0^b), and 4T1 breast cancer (Fig. 10c) cells.

## Discussion

Cancer therapy has historically been categorized by toxicity and various adverse events. The introduction of immunotherapy has offered the possibility of enhanced specificity to cancer cells, thus reducing numerous possible adverse effects. Unfortunately, this does not spare the risk of autoimmunity as a result of excessive immune activation, nor the risk of therapeutic resistance, which occurs in a significant proportion of these patients. In order to develop a novel approach to immunotherapy, we decided to explore the possibility of reducing senescent cell burden by administration of an immunotherapy agent capable of augmenting the body’s ability to remove senescent cells. By leveraging the concept of autologous dendritic cells pulsed with cell lysate, we successfully generated an immunocellular therapeutic that was capable of selectively inducing immunity to senescent, but not control cells. Others have shown ability of cellular lysates to act as polyvalent vaccines. For example, NovaRx, Inc used a combination of three cell lines with antisense to TGF-beta to induce immunity to lung cancer using a product called Lucanix®. They effectively have shown regressions in pre-clinical (murine) studies as well as in early clinical trials [50] but failed in Phase III [51]. CancerVax demonstrated successful induction of immunity in melanoma using irradiated cells. Though positive results were demonstrated in Phase I and II trials [52], their Phase III trial was associated with failure [53]. Despite this, immunity was successfully generated and patients in which strong immunity was identified actually experienced remissions [54]. Northwest Biotherapeutics is currently running a Phase III trial in glioma using patient lysates to pulse autologous dendritic cells, with complete remission seen in many of the studied patients [55–57]. In a study breaking tolerance to tumor associated tissues, Batu Biologics broke tolerance to cancer endothelial cell antigens, resulting in tumor regression in animal models with published human safety [58, 59] as well.

We believe that in contrast to Lucanix or CancerVax, SenoVax possesses enhanced probability of success firstly because dendritic cells are loaded with antigens ex vivo, thus hypothetically increasing immunogenicity. Additionally, while tumor cells contain immune suppressive molecules such as TGF-beta [60, 61], soluble HLA-G [62], and IL-10 [63], intra alia, senescent cell lysates contain molecules that may actually stimulate immunity through dendritic cell maturation such as interleukin-6 [64].

Subsequent to generating a reproducible source of syngeneic senescent cells using dermal fibroblasts as starting material, we proceeded to pulse dendritic cells with their lysate. Establishment of an appropriate protocol for this is critical because cellular lysates could be cytotoxic to dendritic cells, or can induce hypermaturation causing activation induced cell death. The optimized protocol allowed for generation of viable and functional DCs, which possessed similar allo-stimulatory activity as control DCs. Additionally, the “SenoVax™” DCs were demonstrated to possess expression of the p16 protein that they had engulfed.

Immunization with SenoVax™ resulted in both prophylaxis of tumor growth, as well as reduction of size of established tumors. Additionally, SenoVax™ reduced the number of pulmonary metastases when administered intravenously and pulmonary metastases were assessed. We showed that injection with SenoVax™ induced production of antibodies and T cells that were senolytic to senescent cells in vitro, additionally, a time-dependent reduction of the senescent associated markers IL-11, IL-6, IL-23 receptor and YLK-40, was observed only after immunization. While this provides a good surrogate marker, future studies will assess reduction of senescent cell burden by histology and quantification of “senescent infiltrating lymphocytes” in vivo.

Transfer of immunity to naïve recipients was accomplished only with CD8 cell populations, but not CD4 or CD19 cells. This is supported by the in vivo recall studies which showed enhanced expression of Granzyme B, which is associated with T cytotoxic populations. Importantly, our data demonstrated that SenoVax™-induced immunity could be amplified by checkpoint inhibitors and that anticancer activity is not restricted to lung cancer, but also can reduce growth of breast, glioma and pancreatic cancers.

The possibility of using senolytic approaches in oncology lends itself not only towards directly inducing tumor killing but also enhancing responses to conventional cancer therapeutics. For example, Chaib et al. demonstrated that chemotherapy induces senescence in cancer cells, leading to high upregulation of PD-L2, an immune checkpoint protein that enables senescent cells to evade immune detection and persist in tumors. PD-L2 is not essential for senescence induction but is critical for suppressing immune responses, as its deficiency results in rapid clearance of senescent cells, reduced chemokine production (CXCL1/CXCL2), and prevention of myeloid-derived suppressor cell recruitment. Consequently, blocking PD-L2 with antibodies synergizes with chemotherapy to drive CD8 T cell-mediated tumor regression in pancreatic and mammary cancer models, offering a novel therapeutic strategy targeting therapy-induced senescence vulnerabilities [65]. Indeed, we have observed synergy between checkpoint inhibitors and SenoVax. Additionally, preliminary data indicates synergy between chemotherapy and SenoVax (studies in progress).

To our knowledge, this data is the first time that induction of polyvalent responses to senescent cell antigens has been induced. Other previous studies have shown that epitope vaccines, DNA vaccines, and protein vaccines are capable of breaking tolerance to senescent cells and inducing cancer regression. Additionally, CAR-T cell approaches have also been successfully used in this context.

In conclusion, we demonstrate a potentially clinically translatable therapeutic approach for inducing immunity to senescent cells. Given the immunosuppressive, chemoresistant and radioresistant effects of senescent cells, future studies are needed to evaluate synergy of SenoVax™ with existing therapeutic approaches.

## Data Availability

All data generated and analyzed in this study are included in this article.

## References

[1] Sung H, et al. Global cancer statistics 2020: GLOBOCAN estimates of incidence and mortality worldwide for 36 cancers in 185 countries. CA Cancer J Clin. 2021;71(3):209–49 33538338 10.3322/caac.21660

[2] Siegel RL, Miller KD, Jemal A. Cancer statistics, *2020*. CA Cancer J Clin. 2020;70(1):7–30. 31912902 10.3322/caac.21590

[3] Molina JR, et al. Non-small cell lung cancer: epidemiology, risk factors, treatment, and survivorship. Mayo Clin Proc. 2008;83(5):584–94. 18452692 10.4065/83.5.584 PMC2718421

[4] Lofling L, et al. Temporal trends in lung cancer survival: a population-based study. Acta Oncol. 2022;61(5):625–31. 34889167 10.1080/0284186X.2021.2013529

[5] Pao W, Chmielecki J. Rational, biologically based treatment of EGFR-mutant non-small-cell lung cancer. Nat Rev Cancer. 2010;10(11):760–74. 20966921 10.1038/nrc2947 PMC3072803

[6] Rais T, et al. Repotrectinib: a promising new therapy for advanced nonsmall cell lung cancer. Ann Med Surg (Lond). 2024;86(12):7265–69. 39649881 10.1097/MS9.0000000000002717 PMC11623886

[7] Roy M, et al. Nonsmall cell lung cancer therapy: insight into multitargeted small-molecule growth factor receptor inhibitors. Biomed Res Int. 2013;2013:964743. 23936861 10.1155/2013/964743 PMC3713357

[8] Yu L, et al. Targeted therapy of non-small cell lung cancer: mechanisms and clinical trials. Front Oncol. 2024;14:1451230. 39391239 10.3389/fonc.2024.1451230 PMC11464343

[9] Li F, et al. Programmed cell death protein 1/Programmed cell death protein ligand 1 immunosuppressants in advanced non-small cell lung cancer research progress in treatment. Front Pharmacol. 2022;13:918709. 35784705 10.3389/fphar.2022.918709 PMC9243588

[10] Zarour HM. Reversing T-cell dysfunction and exhaustion in cancer. Clin Cancer Res. 2016;22(8):1856–64. 27084739 10.1158/1078-0432.CCR-15-1849 PMC4872712

[11] Belizario J, Destro Rodrigues MF. Checkpoint inhibitor blockade and epigenetic reprogrammability in CD8(+) T-cell activation and exhaustion. Ther Adv Vaccines Immunother. 2020;8:2515135520904238. 32206744 10.1177/2515135520904238 PMC7074507

[12] Balanca CC, et al. PD-1 blockade restores helper activity of tumor-infiltrating, exhausted PD-1hiCD39+ CD4 T cells. JCI Insight. 2021;6(2). 10.1172/jci.insight.142513 PMC7934837 33332284

[13] Siddiqui I, et al. Intratumoral Tcf1(+)PD-1(+)CD8(+) T cells with stem-like properties promote tumor control in response to vaccination and checkpoint blockade immunotherapy. Immunity. 2019;50(1):195–211 e10. 30635237 10.1016/j.immuni.2018.12.021

[14] Sanchez-Paulete AR, et al. Cancer immunotherapy with immunomodulatory anti-CD137 and anti-PD-1 monoclonal antibodies requires BATF3-dependent dendritic cells. Cancer Discov. 2016;6(1):71–79. 26493961 10.1158/2159-8290.CD-15-0510 PMC5036540

[15] Zhou S, Yang H. Immunotherapy resistance in non-small-cell lung cancer: from mechanism to clinical strategies. Front Immunol. 2023;14:1129465. 37090727 10.3389/fimmu.2023.1129465 PMC10115980

[16] Ge J, Yin X, Chen L. Regulatory T cells: masterminds of immune equilibrium and future therapeutic innovations. Front Immunol. 2024;15:1457189. 39290699 10.3389/fimmu.2024.1457189 PMC11405253

[17] Mani NL, et al. Acidity induces durable enhancement of T(reg) cell suppressive functions for tumor immune evasion. Mol Immunol. 2024;174:57–68. 39213947 10.1016/j.molimm.2024.08.004 PMC11681611

[18] Mukherjee S, et al. Breast cancer stem cells generate immune-suppressive T regulatory cells by secreting TGFbeta to evade immune-elimination. Discov Oncol. 2023;14(1):220. 38038865 10.1007/s12672-023-00787-z PMC10692020

[19] Li MY, Ye W, Luo KW. Immunotherapies targeting tumor-associated Macrophages (TAMs) in cancer. Pharmaceutics. 2024;16(7). 10.3390/pharmaceutics16070865 PMC11280177 39065562

[20] Zhang J, et al. Engineering and targeting neutrophils for cancer therapy. Adv Mater. 2024;36(19):e2310318. 38320755 10.1002/adma.202310318

[21] Rajkumari S, et al. Myeloid-derived suppressor cells in cancer: current knowledge and future perspectives. Int Immunopharmacol. 2024;142(Pt A):112949. 10.1016/j.intimp.2024.112949 39236460

[22] Lv K, He T. Cancer-associated fibroblasts: heterogeneity, tumorigenicity and therapeutic targets. Mol Biomed. 2024;5(1):70. 39680287 10.1186/s43556-024-00233-8 PMC11649616

[23] Bellone G, et al. Cooperative induction of a tolerogenic dendritic cell phenotype by cytokines secreted by pancreatic carcinoma cells. The J Immunol. 2006;177(5):3448–60. 16920987 10.4049/jimmunol.177.5.3448

[24] Assouline B, et al. Senescent cancer-associated fibroblasts in pancreatic adenocarcinoma restrict CD8(+) T cell activation and limit responsiveness to immunotherapy in mice. Nat Commun. 2024;15(1):6162. 39039076 10.1038/s41467-024-50441-7 PMC11263607

[25] Ji P, et al. Targeted clearance of senescent cells via engineered extracellular vesicles reprograms tumor immunosuppressive microenvironment. Adv Healthc Mater. 2024;13(23):e2400945. 38794820 10.1002/adhm.202400945

[26] Pessoa J, Nobrega-Pereira S, de Jesus BB. Senescent cell-derived vaccines: a new concept towards an immune response against cancer and aging? Aging (albany NY). 2024;16(12):10657–65. 38942604 10.18632/aging.205975 PMC11236300

[27] Diao L, Liu M. Rethinking antigen source: cancer vaccines based on whole tumor cell/tissue lysate or whole tumor cell. Adv Sci (Weinh). 2023;10(22):e2300121. 37254712 10.1002/advs.202300121 PMC10401146

[28] Nestle FO, et al. Vaccination of melanoma patients with peptide- or tumor lysate-pulsed dendritic cells. Nat Med. 1998;4(3):328–32. 9500607 10.1038/nm0398-328

[29] Holtl L, et al. Cellular and humoral immune responses in patients with metastatic renal cell carcinoma after vaccination with antigen pulsed dendritic cells. J Urol. 1999;161(3):777–82. 10022683

[30] Inoue C, et al. Cancer-associated fibroblast marker signatures and stromal composition in usual interstitial pneumonia-associated lung adenocarcinoma: an analysis using a proteomic-immunohistochemical approach. Virchows Arch. 2025.10.1007/s00428-025-04254-840926109

[31] Huang H, et al. Senescent fibroblasts secrete CTHRC1 to promote cancer stemness in hepatocellular carcinoma. Cell Commun Signal. 2025;23(1):379. 40855439 10.1186/s12964-025-02369-8 PMC12376455

[32] O’Fee K, et al. Targeting Cancer-associated Fibroblasts (CAFs) to optimize radiation responses. Cancer J. 2025;31(4). 10.1097/PPO.0000000000000776 40768309

[33] Ruan G, et al. Cancer-associated fibroblasts: dual roles from senescence sentinels to death regulators and new dimensions in therapy. Front Immunol. 2025;16:1635771. 40755775 10.3389/fimmu.2025.1635771 PMC12313499

[34] Zhang X, et al. The multifaceted contributions of cancer-associated fibroblasts to drug resistance in primary and metastatic tumors. Drug Resist Updat. 2025;82:101273. 40617187 10.1016/j.drup.2025.101273

[35] Liu L, et al. Revealing the role of cancer-associated fibroblast senescence in prognosis and immune landscape in pancreatic cancer. iScience. 2025;28(1):111612. 39834857 10.1016/j.isci.2024.111612 PMC11742819

[36] Ou Z, et al. Hypoxia-induced senescent fibroblasts secrete IGF1 to promote cancer stemness in esophageal squamous cell carcinoma. Cancer Res. 2025;85(6):1064–81. 39661488 10.1158/0008-5472.CAN-24-1185

[37] Yamashita T, et al. Immunotherapy-induced reprogramming of cancer-associated fibroblasts can promote tumor progression. Genes Cells. 2024;29(12):1275–83. 39478306 10.1111/gtc.13177

[38] Jiang H, et al. Resveratrol inhibits pancreatic cancer proliferation and metastasis by depleting senescent tumor-associated fibroblasts. World J Gastrointest Oncol. 2024;16(9):3980–93. 39350997 10.4251/wjgo.v16.i9.3980 PMC11438786

[39] Inoue C, et al. Dipeptidyl peptidase 4-positive cancer-associated fibroblasts enhance lung adenocarcinoma growth. Pathol Res Pract. 2024;260:155418. 38908333 10.1016/j.prp.2024.155418

[40] Ye J, et al. Senescent CAFs mediate immunosuppression and drive breast cancer progression. Cancer Discov. 2024;14(7):1302–23. 38683161 10.1158/2159-8290.CD-23-0426 PMC11216870

[41] Belle JI, et al. Senescence defines a distinct subset of myofibroblasts that orchestrates immunosuppression in pancreatic cancer. Cancer Discov. 2024;14(7):1324–55. 38683144 10.1158/2159-8290.CD-23-0428 PMC12155422

[42] Ruhland MK, et al. Stromal senescence establishes an immunosuppressive microenvironment that drives tumorigenesis. Nat Commun. 2016;7:11762. 27272654 10.1038/ncomms11762 PMC4899869

[43] Ichim TE, Zhong R, Min WP. Prevention of allograft rejection by in vitro generated tolerogenic dendritic cells. Transpl Immunol. 2003;11(3–4):295–306. 12967783 10.1016/S0966-3274(03)00048-0

[44] Kellar A, Egan C, Morris D. Preclinical murine models for lung cancer: clinical trial applications. Biomed Res Int. 2015;2015:621324. 26064932 10.1155/2015/621324 PMC4433653

[45] Herbst RS, Takeuchi H, Teicher BA. Paclitaxel/Carboplatin administration along with antiangiogenic therapy in non-small-cell lung and breast carcinoma models. Cancer Chemother Pharmacol. 1998;41(6):497–504. 9554595 10.1007/s002800050773

[46] Papageorgiou A, et al. Effect of navelbine on inhibition of tumor growth, cellular differentiation and estrogen receptor status on Lewis lung carcinoma. Chemotherapy. 2000;46(3):188–94. 10765034 10.1159/000007277

[47] Qin RS, et al. Enhanced antitumor and anti-angiogenic effects of metronomic vinorelbine combined with endostar on Lewis lung carcinoma. BMC Cancer. 2018;18(1):967. 30305062 10.1186/s12885-018-4738-2 PMC6180630

[48] Zhang HP, et al. Effect of sunitinib combined with ionizing radiation on endothelial cells. J Radiat Res. 2011;52(1):1–8. 21187670 10.1269/jrr.10013

[49] Wang P, et al. Chronopharmacology and mechanism of antitumor effect of erlotinib in Lewis tumor-bearing mice. PLoS One. 2014;9(7):e101720. 25000529 10.1371/journal.pone.0101720 PMC4085002

[50] Nemunaitis J, et al. Phase II study of belagenpumatucel-L, a transforming growth factor beta-2 antisense gene-modified allogeneic tumor cell vaccine in non-small-cell lung cancer. J Clin Oncol. 2006;24(29):4721–30. 16966690 10.1200/JCO.2005.05.5335

[51] https://www.cancernetwork.com/view/ecc-lung-cancer-vaccine-trial-fails-meet-endpoint-shows-benefit-some-patients#

[52] Faries MB, et al. Long-term survival after complete surgical resection and adjuvant immunotherapy for distant melanoma metastases. Ann Surg Oncol. 2017;24(13):3991–4000. 29019177 10.1245/s10434-017-6072-3

[53] Slingluff CL, et al. Multicenter, double-blind, placebo-controlled trial of seviprotimut-L polyvalent melanoma vaccine in patients with post-resection melanoma at high risk of recurrence. J Immunother Cancer. 2021;9(10). 10.1136/jitc-2021-003272 PMC8488725 34599031

[54] Petricciani J, Koren E, Morton D. Analysis of the in vivo proliferative capacity of a whole cell cancer vaccine. Biologicals. 2016;44(2):60–63. 26806533 10.1016/j.biologicals.2015.12.005

[55] Fareed A, et al. Dcvax: a promising advancement in oncology for the treatment of glioblastoma. Rare Tumors. 2023;15:20363613231179541. 37256198 10.1177/20363613231179541 PMC10225952

[56] Wheeler CJ, Black KL. Dcvax-brain and dc vaccines in the treatment of GBM. Expert Opin Investig Drugs. 2009;18(4):509–19. 19335279 10.1517/13543780902841951

[57] Ridolfi L, et al. First step results from a phase ii study of a dendritic cell vaccine in glioblastoma patients (CombiG-vax). Front Immunol. 2024;15:1404861. 39192978 10.3389/fimmu.2024.1404861 PMC11347333

[58] Wagner SC, et al. Safety of targeting tumor endothelial cell antigens. J Transl Med. 2016;14:90. 27071457 10.1186/s12967-016-0842-8 PMC4830034

[59] Wagner SC, et al. Induction and characterization of anti-tumor endothelium immunity elicited by ValloVax therapeutic cancer vaccine. Oncotarget. 2017;8(17):28595–613. 28404894 10.18632/oncotarget.15563 PMC5438675

[60] Lohr J, et al. Effector T-cell infiltration positively impacts survival of glioblastoma patients and is impaired by tumor-derived TGF-beta. Clin Cancer Res. 2011;17(13):4296–308. 21478334 10.1158/1078-0432.CCR-10-2557

[61] Sasaki A, et al. Participation of thrombospondin-1 in the activation of latent transforming growth factor-beta in malignant glioma cells. Neurol Med Chir (tokyo). 2001;41(5):253–58; discussion 258-9. 11396305 10.2176/nmc.41.253

[62] Kluckova K, Durmanova V, Bucova M. Soluble HLA-G, its diagnostic and prognostic value and potential target molecule for future therapy in cancer. Bratisl Lek Listy. 2021;122(9):60–617. 34463104 10.4149/BLL_2021_097

[63] Mittal SK, Roche PA. Suppression of antigen presentation by IL-10. Curr Opin Immunol. 2015;34:22–27. 25597442 10.1016/j.coi.2014.12.009 PMC4444374

[64] Upadhyay P, et al. Mechanistic insights and biomarker discovery in immune cell aging and age-associated diseases. Front Immunol. 2025;16:1637191. 41132677 10.3389/fimmu.2025.1637191 PMC12540116

[65] Chaib S, et al. The efficacy of chemotherapy is limited by intratumoral senescent cells expressing PD-L2. Nat Cancer. 2024;5(3):448–62. 38267628 10.1038/s43018-023-00712-x PMC10965441

